# Hyperammonemia induces glial activation, neuroinflammation and alters neurotransmitter receptors in hippocampus, impairing spatial learning: reversal by sulforaphane

**DOI:** 10.1186/s12974-016-0505-y

**Published:** 2016-02-16

**Authors:** Vicente Hernández-Rabaza, Andrea Cabrera-Pastor, Lucas Taoro-González, Michele Malaguarnera, Ana Agustí, Marta Llansola, Vicente Felipo

**Affiliations:** Laboratorio de Neurobiología, Centro de Investigación Príncipe Felipe, Valencia, Spain

**Keywords:** Hepatic encephalopathy, Microglia activation, NMDA receptors, AMPA receptors, GABA receptors

## Abstract

**Background:**

Patients with liver cirrhosis and minimal hepatic encephalopathy (MHE) show mild cognitive impairment and spatial learning dysfunction. Hyperammonemia acts synergistically with inflammation to induce cognitive impairment in MHE. Hyperammonemia-induced neuroinflammation in hippocampus could contribute to spatial learning impairment in MHE. Two main aims of this work were: (1) to assess whether chronic hyperammonemia increases inflammatory factors in the hippocampus and if this is associated with microglia and/or astrocytes activation and (2) to assess whether hyperammonemia-induced neuroinflammation in the hippocampus is associated with altered membrane expression of glutamate and GABA receptors and spatial learning impairment. There are no specific treatments for cognitive alterations in patients with MHE. A third aim was to assess whether treatment with sulforaphane enhances endogenous the anti-inflammatory system, reduces neuroinflammation in the hippocampus of hyperammonemic rats, and restores spatial learning and if normalization of receptor membrane expression is associated with learning improvement.

**Methods:**

We analyzed the following in control and hyperammonemic rats, treated or not with sulforaphane: (1) microglia and astrocytes activation by immunohistochemistry, (2) markers of pro-inflammatory (M1) (IL-1β, IL-6) and anti-inflammatory (M2) microglia (Arg1, YM-1) by Western blot, (3) membrane expression of GABA, AMPA, and NMDA receptors using the BS3 cross-linker, and (4) spatial learning using the radial maze.

**Results:**

The results reported show that hyperammonemia induces astrocytes and microglia activation in the hippocampus, increasing pro-inflammatory cytokines IL-1β and IL-6. This is associated with altered membrane expression of AMPA, NMDA, and GABA receptors which would be responsible for altered neurotransmission and impairment of spatial learning in the radial maze. Treatment with sulforaphane promotes microglia differentiation from pro-inflammatory M1 to anti-inflammatory M2 phenotype and reduces activation of astrocytes in hyperammonemic rats. This reduces neuroinflammation, normalizes membrane expression of glutamate and GABA receptors, and restores spatial learning in hyperammonemic rats.

**Conclusions:**

Hyperammonemia-induced neuroinflammation impairs glutamatergic and GABAergic neurotransmission by altering membrane expression of glutamate and GABA receptors, resulting in impaired spatial learning. Sulforaphane reverses all these effects. Treatment with sulforaphane could be useful to improve cognitive function in cirrhotic patients with minimal or clinical hepatic encephalopathy.

## Background

Patients with liver cirrhosis and minimal hepatic encephalopathy (MHE) show attention deficits, mild cognitive impairment, and spatial memory dysfunction [1–4]. Hyperammonemia is a main factor that acts synergistically with inflammation to induce cognitive impairment in MHE [5–8]. Inflammation and neuroinflammation also contribute to cognitive and motor deficits in situations such as post-operative cognitive dysfunction, aging, and in some mental (schizophrenia) and neurodegenerative (Alzheimer’s) diseases [9–15].

The mechanisms by which neuroinflammation impairs spatial learning are beginning to be unveiled. Spatial learning is mainly modulated in the hippocampus [16] by mechanisms involving NMDA and AMPA receptors for glutamate [17]. Sustained neuroinflammation in the hippocampus alters membrane expression of glutamate and GABA receptors and impairs spatial learning [18–20].

Animal models of MHE such as rats with portacaval shunts also show neuroinflammation which contributes to their cognitive and motor alterations, including spatial learning impairment [20–24].

Chronic hyperammonemia similar to that present in patients with liver cirrhosis and MHE impairs spatial learning in rats in the absence of liver failure [24]. Hyperammonemia per se is enough to induce neuroinflammation in the cerebellum, the most susceptible region in this model [25]. It has not been assessed whether chronic hyperammonemia per se induces neuroinflammation in the hippocampus or alters membrane expression of glutamate and GABA receptors. Two main aims of this work were: (1) to assess whether chronic hyperammonemia increases inflammatory factors in hippocampus and if this is associated with activation of microglia and/or astrocytes and (2) to assess whether hyperammonemia-induced neuroinflammation in the hippocampus is associated with altered membrane expression of glutamate and GABA receptors and spatial learning impairment.

There are no specific treatments for cognitive alterations in patients with MHE. Current treatments are mainly directed to reduce ammonia levels; however, they are not satisfactory, and new treatments acting on brain targets mediating the cognitive alterations could be more effective [8]. As neuroinflammation mediates cognitive impairment in MHE and other pathological situations (see above), a mechanism to improve cognitive function would be to enhance endogenous anti-inflammatory systems to reduce neuroinflammation. This may be achieved by using sulforaphane, which dissociates Nrf2 from keap-1, promoting its translocation to the nucleus and enhancing antioxidant and anti-inflammatory responses [26–28]. A third aim of this work was to assess whether chronic treatment with sulforaphane reduces neuroinflammation in the hippocampus of hyperammonemic rats and restores spatial learning and if normalization of receptors membrane expression is associated with learning improvement.

## Methods

### Animal model

**Fig. 1:**
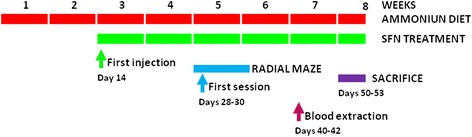
Experimental design

Male Wistar rats were made hyperammonemic by feeding them an ammonium-containing diet as previously described [29] (Fig. 1). The effects of this model of hyperammonemia on body weight and food consumption were reported in detail in [30]. The experiments were approved by the Comite de Experimentación y Bienestar Animal (CEBA) of our Center and performed in accordance with guidelines of the Directive of the European Commission (2010/63/EU) for care and management of experimental animals. Ammonia levels in brain of hyperammonemia rats increase from 166 to 190 % of control rats [25, 31], which is similar to the increase found in models of hepatic encephalopathy such as rats with bile-duct ligation [25, 31].

### Treatment with sulforaphane

Rats were treated daily with sulforaphane (SFN, LKT Laboratory, St. Paul, MN) or saline. Sulforaphane in sterile saline was injected intraperitoneally at 0.5 mg/kg per day. The dose was chosen based on previous studies in the literature [32].

**Fig. 2:**
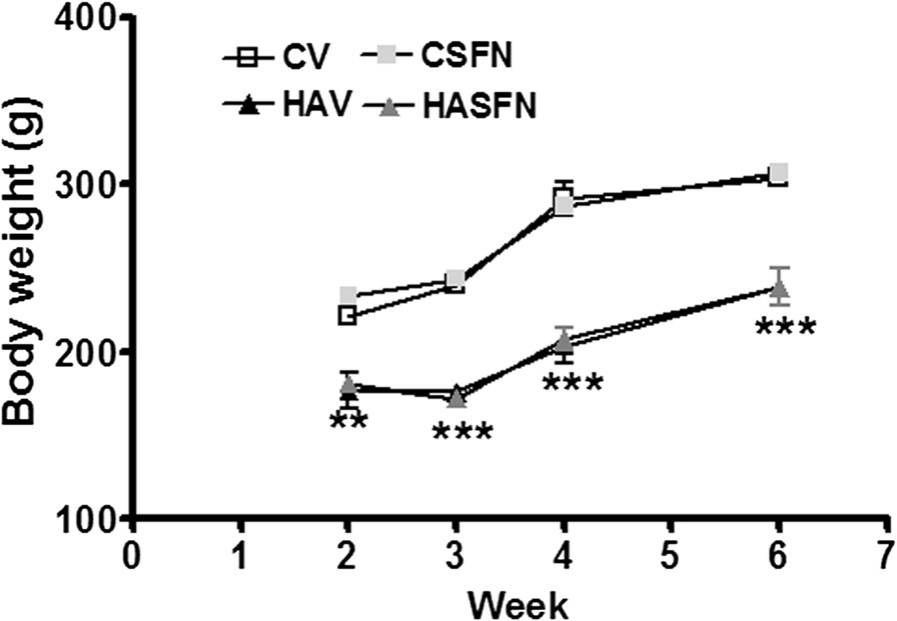
Sulforaphane treatment does not affect body weight. The body weight at 2, 3, 4, and 6 weeks of ammonium diet are shown for control (C) and hyperammonemic (HA) rats treated with vehicle (V) or sulforaphane (SFN). Values are the mean ± SEM of 7 rats per group. Values significantly different from control rats are indicated by *asterisks*. **p < 0.01, ***p < 0.001

Treatment started 2 weeks after the ammonium diet and maintained during all experiments (Fig. 1). Sulforaphane did not affect body weight (Fig. 2) or food consumption in control or hyperammonemic rats.

### Ammonia determination in blood

Blood was taken from the tail vein on days 40–42 after starting the ammonium diet (Fig. 1). Blood ammonia was measured immediately after blood collection with the Ammonia Test Kit II for the PocketChemBA system (Arkay, Inc., Kyoto, Japan) following the manufacturer’s specifications.

### Immunohistochemistry

Coronal 30-μm sections were cut on a cryostat and stored at 4 °C in PB with 0.1 % azide. Free-floating sections were washed; endogenous peroxidase activity was quenched with 3 % H_2_O_2_ for 15 min; and sequential incubations with blocking serum (normal goat serum or horse serum) and primary antibodies (overnight 4 °C) were performed. Primary antibodies were against Iba-1 (1:200) from Abcam (Cambridge, UK) and glial fibrillary acidic protein (GFAP, 1:400) from Sigma (St. Louis, MO). Incubation with biotinylated secondary antibodies and with avidin-biotin-HRP complex (ABC kit, Vector, CA, USA, 1:100) followed. The secondary antibodies (1:200) used were DAB-H_2_O_2_ substrates, used to label antigenic sites. The stained sections were mounted on slides, dehydrated, and coverslipped.

### Analysis of activation of astrocytes and microglia

Analysis of GFAP and Iba-1 staining was performed in the CA1 region of the hippocampus using the ImageJ software. Brain sections from four animals per group were used. Astrocytes and microglia activation was assessed by measuring the cell perimeter in eight randomly selected areas (0.45 mm^2^) per section according to [33]. The perimeter length for each group is expressed as percentage of values for control rats.

### Analysis of proteins content by Western blot

The animals were sacrificed (Fig. 1) by decapitation and the whole hippocampi were dissected and homogenized in 66 mM Tris-HCl (pH 7.4), 1 % SDS, 1 mM EGTA, 10 % glycerol, 1 mM sodium ortho-vanadate, and 1 mM sodium fluoride containing protease inhibitor cocktail (Roche, Mannheim, Germany). Samples were subjected to electrophoresis and immunoblotting as in [34]. Primary antibodies were against Iba-1,Ym-1 (1:2000) from Abcam (Cambridge, UK), IL-1β (1:500) from R&D SYSTEMS, Minneapolis, USA; IL-6 (1:500) from Biosource, Camarillo, USA; IL-4 and IL-10 (1:1000) from Abcam (Cambridge, MA) and TNF-α (1:500) from R&D SYSTEMS (Minneapolis, USA), Arg-1 from BD Bioscience (NJ, USA), and glial fibrillary acidic protein (GFAP) (1:5000) from Sigma (St. Louis, MO, USA). As a control for protein loading, the same membranes were also incubated with anti-actin (Abcam, Cambridge, MA; 1:1,000). Secondary antibodies were anti-rabbit, anti-goat, or anti-mouse IgG (1:2000) conjugated with alkaline phosphatase (Sigma, St. Louis, MO). The images were captured using the ScanJet 5300C (Hewlett- Packard, Amsterdam, The Netherlands) and band intensities quantified using the Alpha Imager 2200, version 3.1.2 (AlphaInnotech Corporation, San Francisco).

### Analysis of membrane surface expression of glutamate and GABA receptors

Membrane surface expression of glutamate and GABA receptors in whole hippocampal slices was analyzed as described by [35], by cross-linking with BS3.

Transverse hippocampal slices (400 μm) were obtained using a manual chopper. Slices were added to Eppendorf tubes containing ice-cold standard buffer with or without 2 mM BS_3_ (Pierce, Rockford, IL). Incubation with gentle agitation proceeded for 30 min at 4 °C. Cross-linking was terminated by quenching the reaction with 100 mM glycine (10 min, 4 °C). Slices were suspended in ice-cold lysis buffer containing protease and phosphatase inhibitors and homogenized rapidly by sonicating for 20 s. Samples treated or not with BS_3_ were analyzed by Western blot using antibodies against AMPA-GluR1 (1:1000, Calbiochem), AMPA-GluR2 (1:1000, Millipore), NMDA-NR1 (1:1000, BD Pharmigen), NMDA-NR2A (1:1000, Millipore), GABA_A_ α1 (1:1000, Abcam), and GABA_A_ α5 (1:500, Abcam). The surface expression of the receptors was calculated as the difference between the intensity of the bands without BS3 (total protein) and with BS3 (non-membrane protein).

### Spatial learning in the 8-arm radial maze

Spatial learning was assessed as described in [36].

After 2 days of pre-training, training was performed during 5 days (five trials per day) (Fig. 1). The task involved locating four pellets, each placed at the end of a different arm according to a random configuration. Configurations were specific for each rat and were kept invariable throughout training. The number of right choices (first visits to baited arms) and spatial learning errors (first visits to un-baited arms) were calculated for each day. A learning index defined as number of right choices-learning errors was used to evaluate learning of the task. A criterion of a learning index was determined in 10 points.

### Statistical analysis

Results are expressed as mean ± SEM. Data were analyzed by analysis of variance (ANOVA). Newman-Keuls multiple post hoc comparisons were made after the ANOVA to explore main and interaction effects. Significance levels were set at *α* < 0.05.

## Results

Blood ammonia levels were 18 ± 3 μm in control rats and were significantly (*p* < 0.001) increased in hyperammonemic rats to 54 ± 7 μm. Sulforaphane did not affect ammonia levels in control (18 ± 3 μm) or hyperammonemic (49 ± 6 μm) rats. These data show that the effects of sulforaphane are not due to reduction of hyperammonemia.

### Hyperammonemic rats show activation of microglia and astrocytes in the hippocampus which is reversed by sulforaphane

**Fig. 3:**
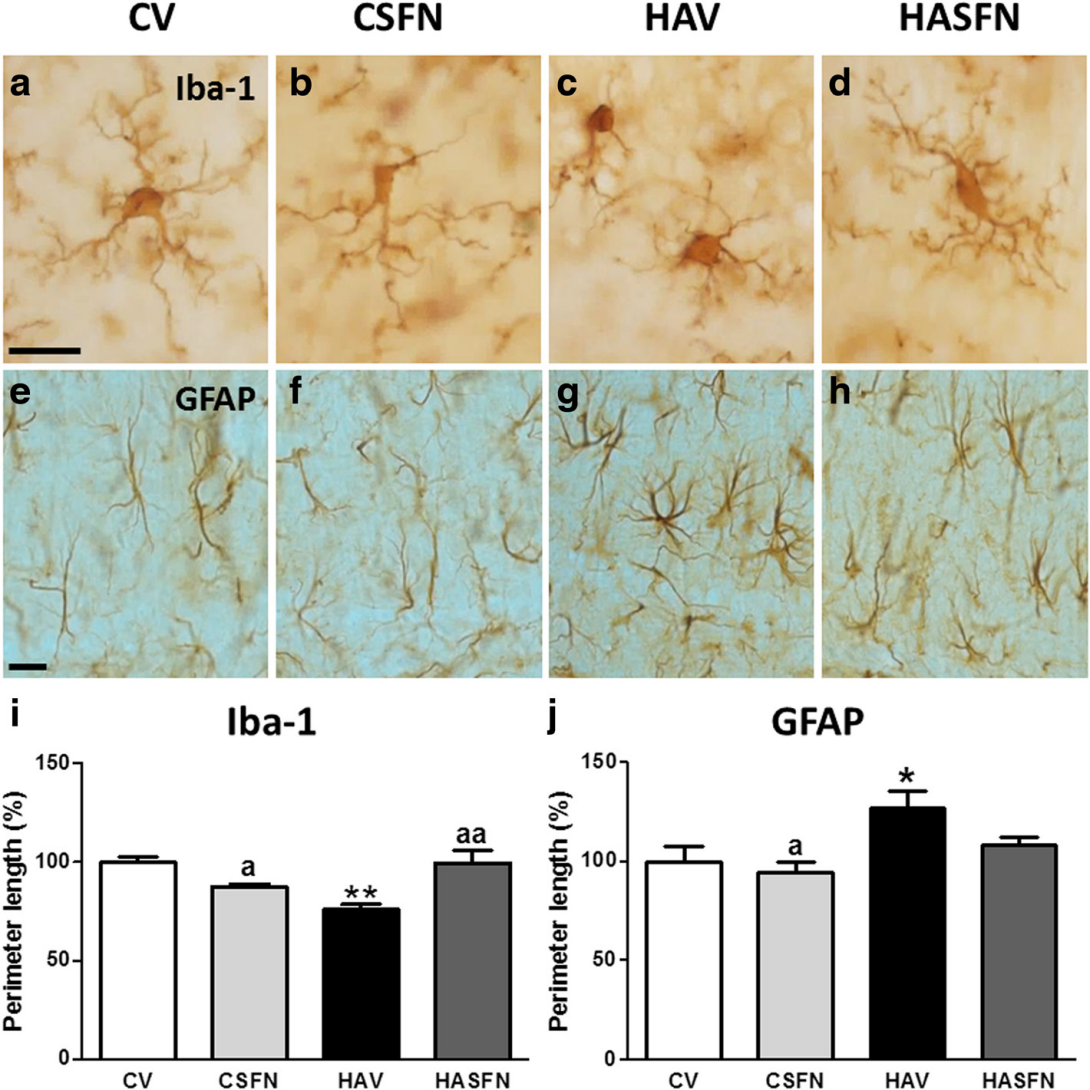
Hyperammonemia induces activation of microglia and astrocytes in the hippocampus, which are reversed by sulforaphane. Activation of microglia and astrocytes in the CA1 region of the hippocampus was assessed by immunohistochemistry in control (C) and hyperammonemic (HA) rats treated with vehicle (V) or sulforaphane (SFN). Microglia and astrocytes were stained with Iba-1 (**a**–**d**) and GFAP (**a**–**h**) antibodies, respectively. **i**–**l** The perimeter of the cells was measured using the ImageJ analysis software after setting an intensity threshold and size filter. The results were expressed as percentage of values in control rats for microglia (**i**) and astrocytes (**j**). Values significantly different from control rats are indicated by *asterisks*. Values significantly different from hyperammonemic rats are indicated by *a*. *p < 0.05, **p < 0.01, ^*a*^p < 0.05, ^*aa*^ *p* < 0.01. *Scale bars* 20 μm

Hyperammonemic rats show activation of microglia in hippocampus, with a significant (*p* < 0.01) reduction of the perimeter length to 78 ± 3 % of control rats. Treatment with sulforaphane restored perimeter length in hyperammonemic rats to 100 ± 3 % of control rats and slightly reduced it (*p* < 0.05) in control rats to 88 ± 2 % of untreated control rats (Fig. 3a–d, i).

Hyperammonemic rats also show astrocytes activation, with altered morphology (Fig. 3g vs. Fig. 3e) and increased perimeter length (Fig. 3j), which increased to 127 ± 9 % of controls (*p* < 0.05). Sulforaphane treatment reduced activation of astrocytes which returned to normal morphology (Fig. 3h vs. Fig. 3g) and to normal perimeter length (127 ± 8 % of controls) (Fig. 3 j).

**Fig. 4:**
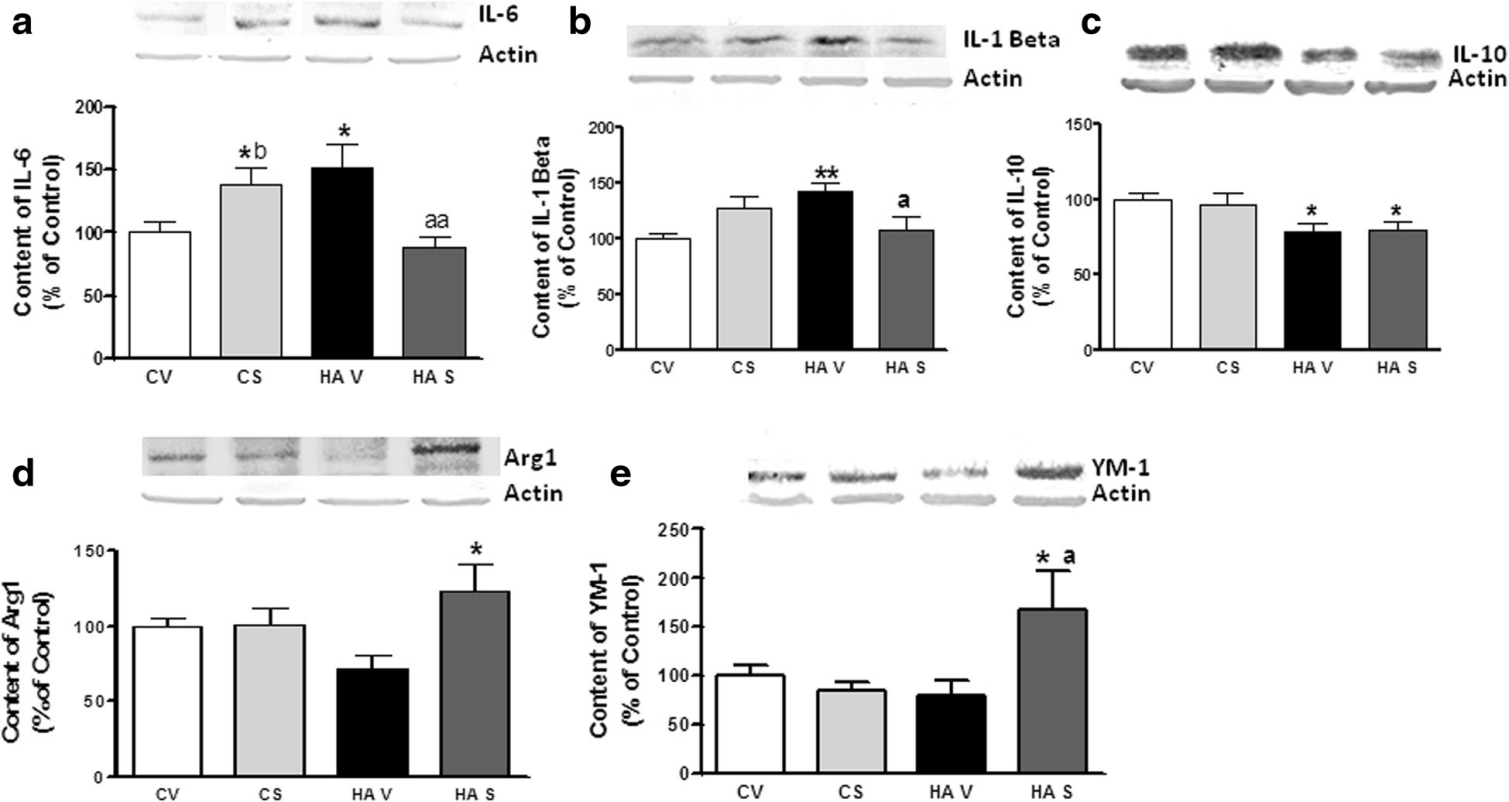
Hyperammonemia increases pro-inflammatory M1 markers IL-1β and IL-6 in the hippocampus. Sulforaphane reduces M1 markers and increases M2 markers Arginase 1 and Ym-1. The hippocampal content of IL-6 (**a**), IL-1β (**b**), IL-10 (**c**), Arginase 1 (**d**), and Ym-1 (**e**) was analyzed by Western blot in control (C) and hyperammonemic (HA) rats treated with vehicle (V) or sulforaphane (SFN). Representative images are shown. Values are the mean ± SEM of 8–12 rats per group. Values significantly different from control rats are indicated by *asterisks*. Values significantly different from hyperammonemic rats are indicated by *a*. *p < 0.05, **p < 0.01, ^*a*^ *p* < 0.05, ^*aa*^ *p* < 0.01

The presence of neuroinflammation was confirmed by analyzing the content of inflammatory markers by western blot in the whole hippocampus (Fig. 4). Hyperammonemic rats show increased levels (*p* < 0.05) of the pro-inflammatory cytokines IL-6 and IL-1β, which reached 151 ± 19 and 142 ± 7 % of controls, respectively. Treatment with sulforaphane normalized the levels of IL-6 and IL-1β, which returned to 88 ± 7 and 106 ± 11 % of controls, respectively (Fig. 4a, b).

TNF-α levels were increased in hyperammonemic rats (115 ± 9 % of controls), but the effect was not statistically significant. Sulforaphane reduced TNF-α in hyperammonemic rats to 94 ± 7 % of controls and increased in ion control rats, non significantly, to 117 ± 7 % of controls. The levels of anti-inflammatory cytokine IL-10 were reduced (*p* < 0.05) in the hippocampus of hyperammonemic rats to 79 ± 6 % of controls and were not normalized by sulforaphane, remaining at 80 ± 7 % of controls (Fig. 4c). The levels of IL-4 were not affected by hyperammonemia (104 ± 8 % of controls) or sulforaphane (87 ± 11 %).

To assess whether the anti-inflammatory effect of sulforaphane is due to promotion of differentiation of microglia from pro-inflammatory M1 to anti-inflammatory M2 phenotype, we analyzed the content of M2 markers. Hyperammonemia tended to reduce the M2 markers Arginase 1 (Arg-1) and Ym-1, but the effects were not statistically significant. Treatment of hyperammonemic rats with sulforaphane increased (*p* < 0.05) the hippocampal levels of Arg-1 and Ym-1 to 123 ± 17 and 168 ± 32 % of controls, respectively. However, sulforaphane did not affect Arg-1 and Ym-1 in control rats (Fig. 4d, e).

### Hyperammonemia alters membrane expression of GABA, AMPA, and NMDA receptors in the hippocampus and sulforaphane reverses these changes

It has been shown that neuroinflammation may affect neurotransmission by altering membrane expression of glutamate and GABA receptors [18–20]. We therefore assessed whether neuroinflammation in the hippocampus of hyperammonemic rats is associated with altered membrane expression of these receptors.

**Fig. 5:**
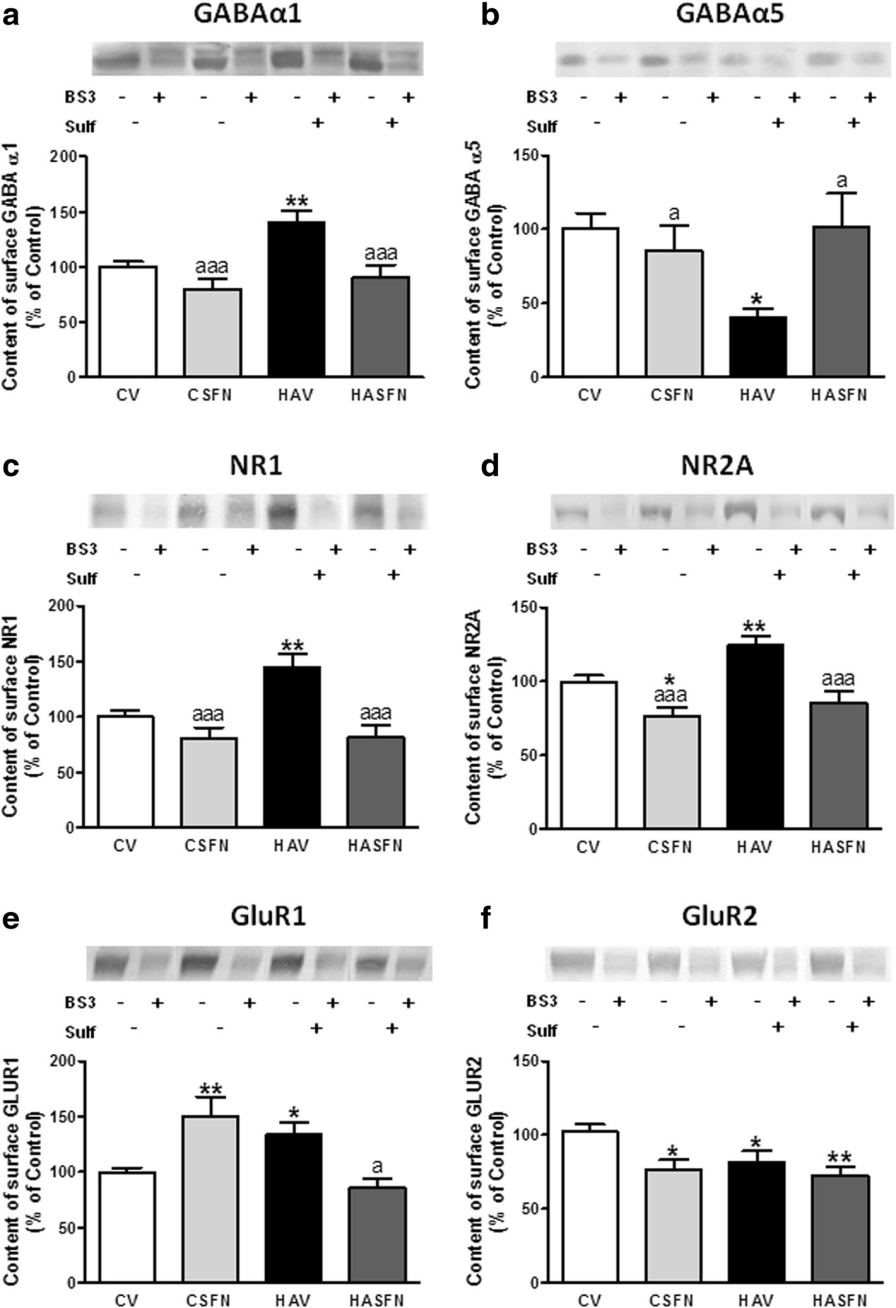
The membrane expression of the alpha-1 (**a**) and alpha-5 (**b**) subunits of GABA_A_ receptor, GluR1 (**e**) and GluR2 (**f**) subunits of AMPA receptors, and NR1 (**c**) and NR2A (**d**) subunits of NMDA receptors is altered in the hippocampus of hyperammonemic rats and is normalized by treatment with sulforaphane. Membrane expression of each subunit in the hippocampus was analyzed using the BS3 cross-linker procedure as described in the Methods section. Samples incubated in the absence or presence of BS3 were subjected to Western blotting using antibodies for each of the subunits. Representative images are shown. Samples in the absence of BS3 represent the total amount of each protein. Samples in the presence of BS3 represent the non-membrane fraction. The intensities of the bands were quantified, and membrane expression was calculated as the difference of intensity between samples without and with BS3. Values are expressed as percentage of control rats and are the mean ± standard errors of 8–12 rats per group. Values significantly different from control rats are indicated by *asterisks* and from hyperammonemic rats by *a*. *p < 0.05,**p < 0.01, ^*a*^ *p* < 0.05, ^*aaa*^ *p* < 0.001

The membrane expression of the alpha-1 subunit of GABA receptors is increased (*p* < 0.01) in hyperammonemic rats to 141 ± 10 % of controls (Fig. 5a) while that of the alpha-5 subunit is reduced (*p* < 0.05) to 40 ± 6 % of controls (Fig. 5b). Sulforaphane reverses these effects, and membrane expression of the alpha-1 and alpha-5 subunits return to 90 ± 12 and 102 ± 22 % of controls, respectively.

The membrane expression of both NR1 (Fig. 5c) and NR2A (Fig. 5d) subunits of NMDA receptors is increased (*p* < 0.01) in hyperammonemic rats to 145 ± 12 and 124 ± 6 % of controls, respectively. Sulforaphane reverses these effects, and membrane expression of the NR1 and NR2A subunits return to 82 ± 11 and 86 ± 8 % of controls, respectively (Figs. 5c and 4d).

Concerning AMPA receptors, the membrane expression of the GluR1 subunit is increased (*p* < 0.05) in hyperammonemic rats to 133 ± 11 % of controls (Fig. 5e) while the expression of the GluR2 subunit is reduced (*p* < 0.05) to 82 ± 7 % of controls (Fig. 5f). Sulforaphane reversed the effects on GluR1, which membrane expression returned to 85 ± 9 % of controls (Fig. 5e), but not the effect on GluR2, which expression remained at 73 ± 6 % of controls (Fig. 5f).

### Hyperammonemia impairs spatial learning in the radial maze, and sulforaphane restores it

We used two parameters to quantify learning ability in the radial maze: the learning index and the trials needed to reach the learning criterium. In control rats, the learning index increased progressively during the 5 days of tests. Control rats improved their performance across the days. In two-way RM ANOVA, the effect of training days was very significant, *p* < 0.001. The difference was significant when comparing day 1 with days 3 and 4 (*p* < 0.01) or with day 5 (*p* < 0.001), also when comparing day 2 with day 5 (*p* < 0.05). In hyperammonemic rats, improvement across the days was lower. The difference was significant only when comparing days 1 or 3 with day 5 (*p* < 0.05).

**Fig. 6:**
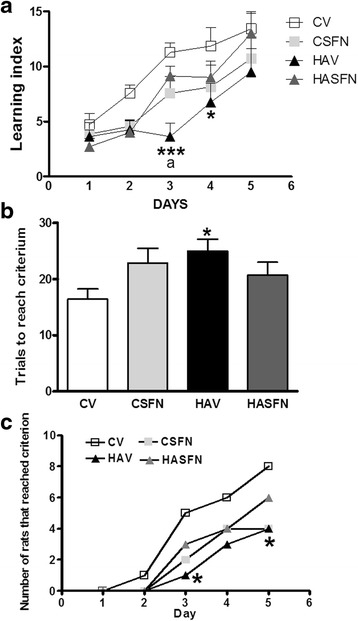
Hyperammonemia impairs and sulforaphane restores spatial learning in the radial maze. Spatial learning ability in the radial maze was assessed in control (C) and hyperammonemic (HA) rats, treated with vehicle (V) or sulforaphane (SFN). Values are the mean ± SEM of seven rats per group. **a** Learning index was calculated as indicated in the Methods section. **b** Shows the number of trails needed to reach a learning criterion set at 10 points of learning index. **c** Shows for each day the number of rats that have reached the criterion. Values significantly different from control rats or hyperammonemic rats are indicated by *asterisks* or *a*, respectively. *p < 0.05,**p < 0.01, ***p < 0.001, ^*aa*^ *p* < 0.01

With two-way ANOVA with repeated measures, the statistics were: interaction effect between treatment and training, *F* = 1.8 and *p* = 0.067; training effect, *F* = 30 and *p* < 0.0001; treatment, *F* = 4.7 and *p* = 0.0098, and matching *F* = 3.5 and *p* < 0.0001. Learning index was significantly lower in hyperammonemic rats at days 3 and 4 (2.7 ± 0.9 and 5.7 ± 1.6, respectively) than that in control rats (11.3 ± 0.9 and 11.9 ± 1.7, respectively). Sulforaphane treatment significantly (*p* < 0.01) improved learning index in hyperammonemic rats to 9.1 ± 0.9 and 9.0 ± 1.5 on days 3 and 4, respectively (Fig. 6a).

Hyperammonemic rats needed more trails (26 ± 2, *p* < 0.05) than controls (16 ± 2) to reach the learning criterion, confirming reduced learning. Sulforaphane improved learning ability in hyperammonemic rats, reducing to 20 ± 2 the number of trials to reach the criterion, which was not different from controls (Fig. 6b).

The reduced learning ability of hyperammonemic rats is also reflected in the lower number of rats reaching the learning criterion along the training days (Fig. 6c). The difference is statistically significant (*p* < 0.05, in two-way ANOVA) on days 3 and 5. Treatment with sulforaphane increased the number of rats reaching the criterion on each day, which was not different from control rats.

## Discussion

**Fig. 7:**
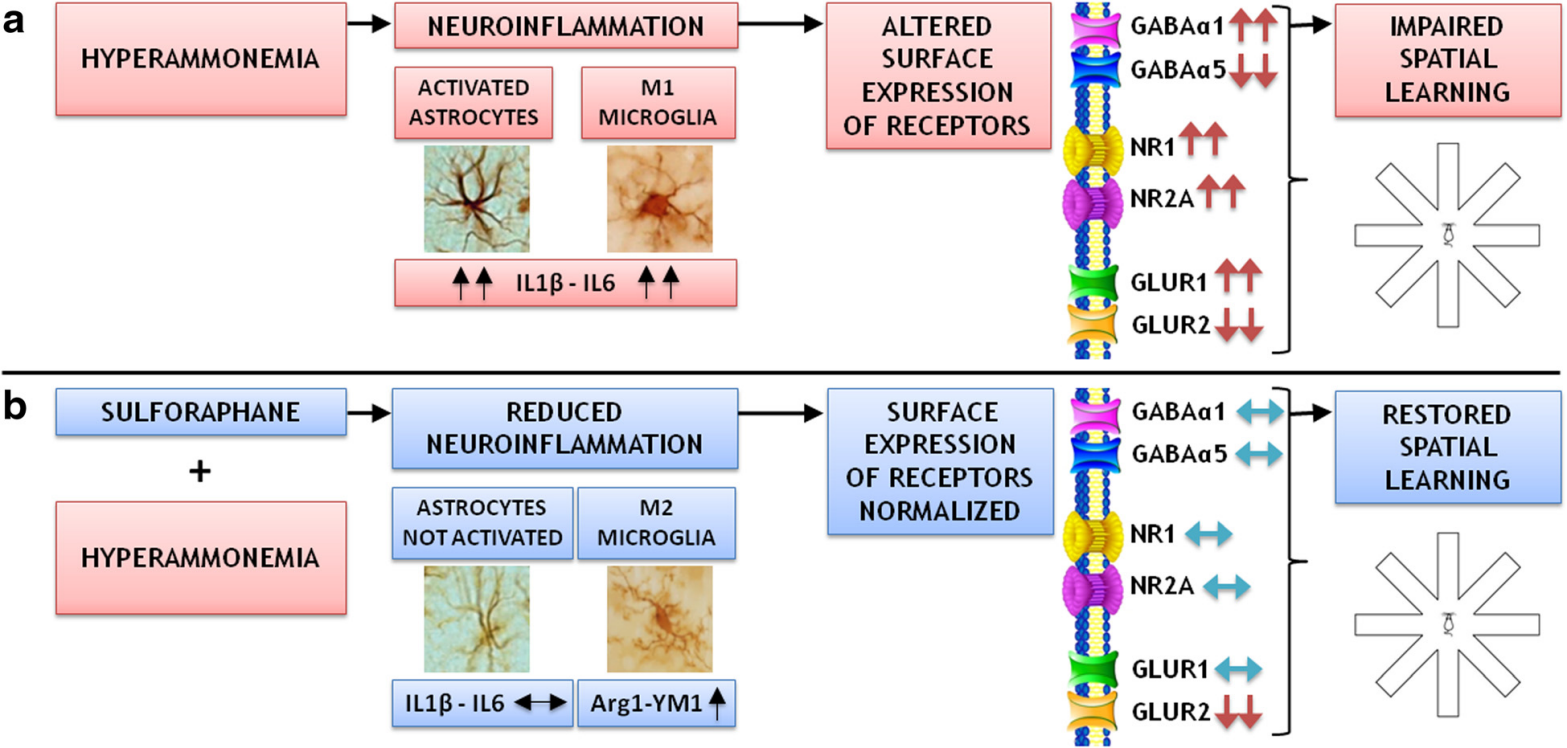
Scheme summarizing the results reported for the effects of hyperammonemia (**a**) and of sulforaphane (**b**). See text for explanation

The data reported are summarized in Fig. 7 and show that hyperammonemia induces activation of astrocytes and microglia in the hippocampus, increasing the levels of pro-inflammatory cytokines IL-1β and IL-6 and reducing the anti-inflammatory IL-10. The levels of TNF-α were increased but not significantly. There was also a tendency to reduce the levels of the M2 microglia markers Arg 1 and Ym-1 which did not reach statistical significance. These data clearly show that hyperammonemia promotes M1 microglia activation and neuroinflammation. The fact that the changes in some markers reach statistical significance while others did not suggest that hyperammonemia induces stronger effects on some pro-inflammatory mechanisms (e.g., activation of M1 microglia) than on others. The milder effects could be masked by the variability of the data, not reaching statistical significance.

This is associated with altered membrane expression of AMPA, NMDA, and GABA receptors which would be responsible for altered neurotransmission and impairment of spatial learning in the radial maze.

Sustained inflammation in the hippocampus impairs spatial learning in different situations including post-operative cognitive dysfunction [37], rats injected with the bacillus Calmette-Guérin in the hippocampus [38] or with lipopolysaccharide [39], and rats with hepatic encephalopathy [20]. Altered long-term potentiation (LTP) in the hippocampus would mediate spatial learning impairment [22].

It is considered that LTP in hippocampus is the basis for spatial learning [40], and the main form of LTP is a consequence of an increased membrane expression of AMPA receptors triggered by activation of NMDA receptors [41]. The mechanism linking glial activation and neuroinflammation with impaired spatial learning would be an altered LTP in the hippocampus as a consequence of the altered membrane expression of AMPA, NMDA, and GABA receptors. IL-1β in the hippocampus impairs LTP [42, 43], and this could be due to altered membrane expression of glutamate and GABA receptors. IL-1β alters membrane expression of GABA_A_ receptors [19, 44] and GluR1 receptors [45].

We show here that in hyperammonemic rats, there is a strong alteration in the membrane expression of GABA, AMPA, and NMDA receptors, with selective increases of alpha-1 subunit of GABA receptors, NR1 and NR2A subunits of NMDA receptors and GluR1 subunit of AMPA receptors, and reduced membrane expression of alpha-5 subunit of GABA receptors and GluR2 subunit of AMPA receptors. This must result in significant alterations in neurotransmission which would contribute to the altered LTP reported for rats with chronic hyperammonemia [46, 47]. Impaired LTP would, in turn, contribute to impair spatial learning in the radial maze.

In this work, we have used a model of chronic hyperammonemia without liver failure. It has been shown that most effects induced by chronic hyperammonemia (including neuroinflammation) are also present in rats with liver failure (e.g., [25]). Hyperammonemia plays an important role in HE; however, other factors are also involved [5–7]. It would be therefore useful to repeat these studies in an animal model with liver failure (such as rats with bile-duct ligation).

Treatment with sulforaphane promotes in hyperammonemic rats the differentiation of microglia from the pro-inflammatory M1 to the anti-inflammatory M2 phenotype, increasing the levels of the M2 markers Arg-1 and Ym-1. This effect is not observed in controls. This may be attributed to the fact that in control rats, microglia is not activated, remaining in resting state, not reaching M1 phenotype. It is not possible therefore to promote its differentiation from M1 to M2. In control rats, sulforaphane increases IL-6 levels while in hyperammonemic rats reduces them. As discussed above, the reduction in hyperammonemic rats would be due to the promotion of microglia differentiation from M1 to M2 phenotype. In control rats, the increase of IL-6 would be a consequence of Nrf2 activation by sulforaphane. It has been reported that the promoter for IL-6 contains a functional antioxidant response element which is activated by Nrf2 and that Nrf2 is a potent activator of IL-6 gene transcription in vivo [48]. Sulforaphane did not restore the levels of IL-10 in hyperammonemic rats, suggesting that a minor part of the effects of hyperammonemia cannot be restored by sulforaphane or that this restoration may take a longer time.

Sulforaphane also reduces astrocyte activation in hyperammonemic rats. This may be due to a direct effect of sulforaphane on astrocytes. It is also possible that astrocyte activation could be a consequence of microglial activation. If this were the case, sulforaphane would be reducing astrocyte activation indirectly, by promoting differentiation of activated microglia to the anti-inflammatory form M2. It has been shown that sulforaphane crosses the blood-brain barrier (reviewed in [49]). It is therefore very likely that the effects of sulforaphane would be due to a direct action on hippocampal microglia and/or astrocytes.

Sulforaphane has been proposed to be beneficial to treat cancer and enhance the anti-tumor activity of cancer therapies [50–52]. The main mechanism by which sulforaphane exerts this beneficial effect was traditionally thought to be through Nrf2-mediated induction of phase 2 detoxification enzymes. However, it is becoming clear that multiple mechanisms activated in response to sulforaphane contribute to its chemoprotective action, including suppression of cytochrome P450 enzymes, induction of apoptotic pathways, suppression of cell cycle progression, inhibition of angiogenesis, and anti-inflammatory activity [50, 51].

Sulforaphane exerts anti-inflammatory effects in some pathological situations such as lipopolysaccharide-induced lung injury [53], experimental autoimmune encephalomyelitis [54], kainate-induced hippocampal cell death [55], and experimental parkinsonism in mice [56]. The mechanisms by which sulforaphane reduces inflammation involves both Nrf2-dependent [53] and independent [57] mechanisms. At the peripheral level, sulforaphane protects from T cell-mediated autoimmune disease by inhibition of IL-23 and IL-12 in dendritic cells and antagonizing Th17-related inflammation in mice [54, 58]. At the brain level sulforaphane activates Nrf2 and antioxidant phase II genes and heme oxygenase [26, 56].

Here, we show that sulforaphane also reduces microglial activation and pro-inflammatory factors in hippocampus by an additional mechanism: promoting differentiation of microglia from the pro-inflammatory M1 to the anti-inflammatory M2 phenotype.

## Conclusions

The results reported show that hyperammonemia induces activation of astrocytes and microglia in the hippocampus, increasing the levels of pro-inflammatory cytokines IL-1β and IL-6. This is associated with altered membrane expression of AMPA, NMDA, and GABA receptors which would be responsible for altered neurotransmission and impairment of spatial learning in the radial maze. Treatment with sulforaphane promotes differentiation of microglia from the pro-inflammatory M1 to the anti-inflammatory M2 phenotype and reduces activation of astrocytes in hyperammonemic rats. This reduces neuroinflammation, normalizes the membrane expression of glutamate and GABA receptors in hippocampus, and restores spatial learning ability in hyperammonemic rats. Treatment with sulforaphane could be useful to improve cognitive function in cirrhotic patients with minimal or clinical hepatic encephalopathy.

## Abbreviations

LTP: long-term potentiation
MHE: minimal hepatic encephalopathy
